# Urinary Concentrations of Steroids in Bulls under Anabolic Treatment by Revalor-XS® Implant

**DOI:** 10.1155/2016/8013175

**Published:** 2016-10-20

**Authors:** Giancarlo Biancotto, Roberto Stella, Federica Barrucci, Francesca Lega, Roberto Angeletti

**Affiliations:** ^1^Department of Chemistry, Istituto Zooprofilattico Sperimentale delle Venezie, Viale dell'Università 10, 35020 Legnaro, Italy; ^2^Department of Public Health and Risk Analysis, Istituto Zooprofilattico Sperimentale delle Venezie, Viale dell'Università 10, 35020 Legnaro, Italy

## Abstract

Despite the European ban of using anabolics in food-producing animals, growth promoters might still be illegally used in the European Union. To control the food chain and guarantee consumers' health, there is a need of highly sensitive analytical methods for the identification of marker residues of such treatments. In the present study, a group of bulls (*n* = 16) received trenbolone acetate (200 mg) and estradiol (40 mg) by a commercial ear implant during a time range of 71 days, and a second group (*n* = 16) was kept for control. The aim of the research was to measure the residual urinary concentrations of the administered drugs (*β*-trenbolone and *β*-estradiol), their main metabolites (*α*-trenbolone and *α*-estradiol), and possible alterations of the urinary profile of other endogenous hormones metabolically related. The analytical method was based on liquid chromatography-tandem mass spectrometry. Results showed average urinary concentrations of *α*-trenbolone and *α*-estradiol during treatment in the range of (0.81 ÷ 2.1) ng mL^−1^ and (0.96 ÷ 4.4) ng mL^−1^, respectively, whereas *β*-trenbolone and *β*-estradiol exhibit urinary concentrations lower than 0.22 ng mL^−1^ in both cases. Data obtained from the urinary profiles of endogenous steroids indicate that they could be useful to indirectly detect the ongoing treatment.

## 1. Introduction

Anabolic steroids, both natural and synthetic, have been used over the last past decades in animal breeding to improve animal growth and feed conversion efficiency [1–4]. Steroid hormones are molecules included in the classes of estrogens, gestagens, and androgens. In some countries, such as the United States, the use of such hormonal growth promoters is still allowed. In contrast, they are banned in food-producing animals in Europe [5]. To enforce the prohibition on the illegal use of anabolic steroids, European Union (EU) member states are required to monitor animals for possible abuse [6]. However, the possibility of abuse of hormonal substances in Europe still exists, mainly due to the economic benefits that these substances provide in animal breeding [7]. The analysis of the drug residues in animals intended for human consumption is probably one of the most challenging tasks in the national monitoring plans of EU members, involving a wide number of substances with different chemical structures and concentration levels in a variety of biological matrices [8–11]. For this reason, several analytical methods have been developed for the determination of hormone residues or their metabolites in samples of animal origin [12–14]. Efficient control of the illegal use of anabolic steroids must rely on the direct measurement of steroid residues, but also on their direct metabolites to take into account kinetics of elimination and metabolic patterns [15–20]. Gas chromatography-mass spectrometry (GC-MS) has played the dominant role for the detection of anabolic steroids in biological samples for many years [21–23]. In recent years, thanks to the rapid advancement of liquid chromatography-tandem mass spectrometry (LC-MS/MS) that has led to an increased sensitivity improving the overall performance, there has been a shift towards LC-MS/MS for the determination of hormones in different matrices [21, 22, 24, 25].

Beside the residue analyses, new approaches have been proposed in recent years to prove illicit treatment with growth promoters [26]: some focused on targeted metabolite analyses [17, 18, 27] and others on untargeted proteomics [28–30] or metabolomics approaches [31–33]. Despite all being promising, none of them is definitive to establish a unique and robust set of biomarkers of treatment independently from drug administration, breed, gender, age, and physiological condition. Still, these studies represent a valuable contribution in the area of research aimed at improving the efficacy of controls against illegal practices with growth promoters.

Moreover, formulations adopted for illegal purposes are not revealed by producers or farmers. Consequently, several research studies have been implemented through the years to test the effect of different growth promoters administered via different routes [18, 28, 34, 35] and to evaluate residual levels and target metabolites. In this work a commercially available ear implant containing trenbolone acetate and estradiol (Revalor-XS) was tested; this formulation is used in the United States in bovine breeding and is able to release a constant amount of hormones over a time period up to 200 days eliminating the need to reimplant the device. The pharmacological trial lasted for 71 days; urine samples were collected before and during the treatment from 16 animals with implant and from 16 untreated animals (control). Depletion studies after treatment suspension were not performed.

The results of this work represent the third pillar of a multitarget research project aimed at verifying the potentiality of the combination of residue analysis, proteomics [29], and metabolomics [33, 36] to control and prevent illicit steroid treatments.

Therefore, the present study provides an analytical evaluation of

- the urinary excretion of the administered drugs and their main metabolites (alpha epimers) after the exogenous administration of trenbolone and estradiol in bulls, introducing useful data regarding their residual concentrations in urine during the treatment period;
- the average urinary concentration of other related steroids physiologically present in the animals, that is, estrone (E1), 17*α*-testosterone (17*α*-T), 17*β*-testosterone (17*β*-T), androstenedione (AED), dihydrotestosterone (DHT), dehydroepiandrosterone (DHEA), and etiocholanolone (ETIO), before, in the middle, and at the end of the trial to verify a possible alteration in the profile of the selected endogenous steroids; in addition, the urinary profiles of endogenous steroids of treated samples were compared to those of control animals using a logistic regression model.

Urine samples were processed using a purification method based on a solid phase extraction (SPE) cleanup common to all target analytes, but with different LC-MS/MS conditions that were optimized for clusters of compounds. The methods were validated following the criteria of Commission Decision 657/2002/EC, properly adapted for our purposes [37].

## 2. Experimental

### 2.1. Reagents and Chemicals

Water was purified and deionized by an ultrapure water system (Sartorius, Stedim Biotech, Aubagne Cedex, France); methanol and acetonitrile (HPLC grade) were from BDH (VWR, West Chester, PA, USA). Formic acid and glacial acetic acid were from Fluka (Buchs, Switzerland); ethyl acetate and sulfuric acid were from Carlo Erba (Milan, Italy). Activated charcoal, dextran, ammonium hydroxide, and *β*-glucuronidase (98,000 U mL^−1^) were from* Helix pomatia* and sulfatase type H2 (2,000 U mL^−1^) was from* Helix pomatia*; *β*-glucuronidase type IX-A was from* E. coli* (12,500 U mL^−1^); filter paper (Whatman, black ribbon) was obtained from Sigma Aldrich (St. Louis, MO, USA). Oasis-HLB reversed-phase SPE cartridges (200 mg, 6 mL) were from Waters (Milford, MA, USA); Strata-NH_2_ SPE cartridges (100 mg, 1 mL) and C_18_ SPE cartridges (500 mg, 6 mL) were obtained from Phenomenex (Torrance, CA, USA). D2-17*β*-testosterone (D2-17*β*-T), D4-17*β*-estradiol (D4-17*β*-E2), and D4-estrone (D4-E1) were from CDN ISOTOPES (Pointe-Claire, Quebec, Canada), D3-dihydrotestosterone (D3-DHT), D2-dehydroepiandrosterone (D2-DHEA), and D7-4-androstene-3,17-dione (D7-AED) were from Steraloids (Newport, RI, USA), and D3-17*β*-trenbolone (D3-17*β*-TBOH) was purchased from RIKILT (Wageningen, Netherlands). 17*β*-Testosterone (17*β*-T), dihydrotestosterone (DHT), 4-androstene-3,17-dione (AED), dehydroepiandrosterone (DHEA), etiocholanolone (ETIO), estrone (E1), 17*α*-estradiol (17*α*-E2), and 17*β*-estradiol (17*β*-E2) were from Sigma Aldrich (St. Louis, MO, USA), 17*α*-trenbolone (17*α*-TBOH) was from Toronto Research Chemicals (Toronto, Ontario, Canada), and 17*β*-trenbolone (17*β*-TBOH) and 17*α*-testosterone (17*α*-T) were purchased from Steraloids (Newport, RI, USA). Primary stock standard solutions of each analyte were prepared in methanol at a concentration of 1000 *μ*g mL^−1^. Intermediate single standard solutions for each analyte were prepared in methanol at a concentration of 10 *μ*g mL^−1^. An internal standard solution which included each deuterated compound was prepared in methanol at a concentration of 0.3 *μ*g mL^−1^. A mixed standard solution was prepared in methanol, with the endogenous compounds at a concentration of 0.05 *μ*g mL^−1^ for E1, 0.125 *μ*g mL^−1^ for AED and 17*α*-E2, 1 *μ*g mL^−1^ for 17*β*-T, 1.25 *μ*g mL^−1^ for DHT and DHEA, 2.5 *μ*g mL^−1^ for 17*α*-T, and 12.5 *μ*g mL^−1^ for ETIO. Finally a mixed standard solution was prepared in methanol for the exogenous substances (i.e., 17*α*-TBOH, 17*β*-TBOH, and 17*β*-E2) at a concentration of 0.125 *μ*g mL^−1^ each. All standards were kept at −20°C.

### 2.2. Sampling and Animal Treatment

32 Charolais bulls at 10–14 months of age were housed in stables and fed for two weeks (acclimatization) after which they were divided into two groups consisting of 16 animals each, and the trial began. Animals owing to the treatment group (T) were implanted using a commercially available device, Revalor-XS (Merck Animal Health), containing 200 mg of TBA and 40 mg of E2. Animals of the control group (C) were not implanted. The trial lasted for 71 days after which animals were slaughtered. Urine samples were collected the day before the beginning of the trial (*T*_0_), 8 times during the trial (*T*_7_, *T*_21_, *T*_28_, *T*_35_, *T*_42_, *T*_49_, *T*_56_, and *T*_63_) and at *T*_68_. Samples were frozen and stored at −20°C until analysis. The animals were managed in agreement with the European Directive 86/609/EEC regarding the protection of animals used for experimental or other scientific purposes, enforced by the Italian D.Lgs 116 (January 27, 1992), and with the Directive 2010/63/UE [38–40]. During the experimentation, hay and water were available to animals* ad libitum*.

### 2.3. Sample Preparation

Urine samples were clarified by centrifugation (5000 ×g for 5 minutes). For each sampling day 2 aliquots (5 mL each) from each animal were analyzed to achieve analyte quantification in duplicate. 50 *μ*L of internal standards containing D3-17*β*-TBOH, D2-17*β*-T, D4-17*β*-E2, D4-E1, D3-DHT, D7-AED, and D2-DHEA at a concentration of 0.3 *μ*g mL^−1^ was added to each vial to obtain a final concentration of 3 *μ*g L^−1^. Depending on the group of analytes, the prepared urine samples were processed using one of the two alternative deconjugation approaches described in the next sections, followed by a common purification protocol.

#### 2.3.1. 17*α*-TBOH, 17*β*-TBOH, 17*α*-E2, and 17*β*-E2 Deconjugation by Enzymes from* Helix pomatia*

The pH was adjusted to 5.0 by adding 15 mL of acetate buffer 0.2 M, pH 5.0, and, if needed, few droplets of HCl 6 N. The hydrolysis was carried out for 2 h at 55°C using 50 *μ*L of *β*-glucuronidase and 50 *μ*L of sulfatase from* Helix pomatia*.

#### 2.3.2. E1, DHT, DHEA, AED, 17*β*-T, 17*α*-T, and ETIO Solvolysis and Deconjugation by *β*-Glucuronidase from* E. coli*

The pH was adjusted to 7.0 by adding 15 mL of phosphate buffer 0.2 M, pH 7.0. The hydrolysis of glucuronide conjugates was carried out for 2 h at 55°C using 50 *μ*L of *β*-glucuronidase from* E. coli*. After deconjugation, samples were cooled at room temperature and the solution was applied to HLB SPE cartridges (200 mg, 6 mL) already activated using 5 mL of methanol and conditioned with 5 mL of water. The column was washed with 5 mL of water, vacuum was applied to dry cartridges, and steroids were eluted using 2 mL of methanol followed by 2 mL of ethyl acetate. The final volume was increased adding ethyl acetate and sulfuric acid (50% in water) to a final volume of 10 mL to obtain a solution containing ethyl acetate/methanol/sulfuric acid (80/20/0.06, v/v/v). The solvolysis of sulfates was carried out for 2 h at 55°C mixing by vortex every 30 min. Samples were neutralized with 60 *μ*L of 2 M NaOH and the volume was decreased to 2 mL under a stream of nitrogen at 45°C. The volume was then increased to 20 mL adding water to reduce the concentration of organic solvent for the subsequent purification step.

#### 2.3.3. Purification of Steroids

After hydrolysis, samples were cooled at room temperature and the solution was loaded onto HLB SPE cartridges (200 mg, 6 mL) previously activated with 5 mL of methanol and conditioned with 5 mL of water. The cartridge was washed with 5 mL of water and 5 mL of methanol/water (50/50, v/v), vacuum was applied, and then analytes were eluted with 4 mL of ethyl acetate/methanol (50/50, v/v). The eluted solution was filtered through a NH_2_ SPE cartridge (100 mg, 1 mL) previously conditioned with 1 mL of methanol and the purified extracts were collected directly during loading. The collected solution was evaporated at 45°C, and steroid extracts were reconstituted in 0.5 mL of water/acetonitrile (70/30, v/v) and analyzed by LC-MS/MS.

### 2.4. Blank Urine Preparation for Quantification of Endogenous Steroids

To remove endogenous steroid compounds from urine used for matrix-matched calibration and quality control samples, a pool of urine samples (250 mL) from veal calves (naturally containing lower amounts of the target analytes) were stirred in presence of charcoal (1.25 g) and dextran (0.125 g) overnight at room temperature, adjusting the method proposed by AbuRuz et al. [41]. Urine samples were finally centrifuged at 5000 ×g for 5 minutes to remove the charcoal in suspension. Afterwards, the charcoal-depleted urine samples were filtered with filter paper, and 5 mL aliquots were stored at −20°C until analysis. Absence of interfering compounds was checked by analyzing an aliquot of blank urine samples during each sample batch.

### 2.5. Analysis by LC-MS/MS

Analysis of steroid residues was achieved using a Prominence HPLC system (Shimadzu, Kyoto, Japan) interfaced to an API 4000 triple quadrupole mass spectrometer with a turbo ion spray source (AB SCIEX, Framingham, MA, USA). Sample extracts were analyzed using 3 different ionization and gradient elution conditions: atmospheric pressure positive chemical ionization (APCI+), positive electrospray (ESI+), and negative electrospray (ESI−). Air was used as nebulization and desolvation gas, while nitrogen was used as curtain gas. Source temperature was set to 550°C with an ion spray voltage of 5500 and −4500 for ESI+ and ESI−, respectively, and 375°C with nebulizer current set at 5 for APCI+. MS/MS experiments were performed using nitrogen as the collision gas at a pressure of 3.5*∗*10^−5^ torr. Ion acquisition was performed in selected reaction monitoring (SRM) using the transitions from the precursor ion to the two most abundant product ions optimizing the declustering potential and the collision energy for each product ion (Table 1).

Purified extracts were separated by reversed-phase liquid chromatography using an Accucore C_18_ column (100 mm × 2.1 mm, 2.6 *μ*m, 100 Å) from Superchrom (Milan, Italy). Mobile phases were water (A1) and acetonitrile (B1) for APCI+, 0.02% (v/v) ammonium hydroxide in water (A2) and acetonitrile/methanol (70/30, v/v) (B2) for ESI+, and 0.02% (v/v) ammonium hydroxide in water (A3) and 0.02% (v/v) ammonium hydroxide in acetonitrile (B3) for ESI−. The different gradients optimized for chromatographic separations are reported in Table 2. The flow rate for the different gradient elution conditions was set to 0.250 mL min^−1^ and the injected volume was 10 *μ*L.

### 2.6. Data Processing and Statistical Analysis

Steroids quantification was performed using MultiQuant software version 2.1 (AB SCIEX, Framingham, MA, USA) integrating the area corresponding to the chromatographic peak of each hormone and the corresponding internal standard. The urinary concentration of exogenous steroids (17*α*-TBOH, 17*β*-TBOH, and 17*β*-E2) was calculated using a matrix-matched calibration curve constructed in urine, while for endogenous steroids (E1, 17*α*-E2, DHT, DHEA, AED, 17*β*-T, 17*α*-T, and ETIO) the curve was constructed in urine previously treated with charcoal to remove endogenous compounds physiologically present. In each batch of analysis the curve was injected at the beginning of the sequence and two fortified samples at the end for quality control purposes. Samples that were fortified with exogenous substances were prepared in urine, while the fortified samples for endogenous steroids were prepared in urine depleted with charcoal.

Steroid concentrations are reported as mean ± standard error of the mean (SEM). The methods used for hormone quantification were validated according to 2002/657/EC Commission Decision.

The difference in measured variables between C and T groups was assessed using Student's unpaired *t*-test, with *p* value < 0.05 considered as statistically significant. In addition a statistical analysis based on a logistic regression model was performed using the statistical software R 2.13.2 (the R Foundation for Statistical Computing, 2011). Data on endogenous steroids profiles of the two groups measured at times *T*_0_, *T*_35_, and *T*_68_ (before, in the middle, and at the end of the treatment) using solvolysis and deconjugation with *β*-glucuronidase from* E. coli* were first standardized by transforming data to have zero mean and unit variance. Then standardized endogenous steroid profiles were used as predictors to infer the probability of belonging to the treated group, using a logit transform of the probability. Finally, the predictive ability of the model was assessed computing the ROC curve.

## 3. Results and Discussion

### 3.1. Selected Treatment

The majority of the studies dealing with sexual steroids administration for growth promotion were performed using a combination of androgens and estrogens administered by single or multiple injections [28, 42–44]. Given the lack of official information regarding the illicit practices employed in livestock production, different drug administration schedules have been tested [19, 35]. Nonetheless, a variety of variables such as the choice of the compounds, dosage, medium for drug resuspension, route, duration, and timing of administration can negatively affect a well-designed trial. For the mentioned reasons, in our study, animals were treated using a device named Revalor-XS, because it is a commercial formulation regularly used in the USA and could therefore be easily available on the market; furthermore the usage and the effectiveness on the feed conversion index are known. This ear implant device is made of coated and uncoated pellets containing 200 mg of TBA and 40 mg of E2 that are continuously released. According to the manufacturers' instructions, four uncoated pellets can immediately release both steroids for about 70 days, and, around that time, the remaining coated pellets begin to deliver their content into the bloodstream for up to 200 days. In this study, for practical and economic reasons, the treatment could last for 71 days, after which animals were sacrificed, preventing us from performing a drug excretion profiling over the remaining ca. 130 days, and a following desirable depletion study. Still, achieved results offer a clear dataset of urinary concentrations of precursors and metabolites during a treatment mimicking an illicit administration for anabolic purposes, typically lasting few weeks. The aim was to provide data regarding the urinary concentration of the administered drugs (17*β*-TBOH and 17*β*-E2) and their main metabolites (17*α*-TBOH and 17*α*-E2) to help understanding the expected residual levels in case of illicit employment of such formulation. Therefore, the extraction/purification protocol was adjusted specifically for these 4 molecules. Moreover, the resulting procedure was slightly modified in the hydrolysis step, for the determination of other 7 endogenous steroids that were analyzed before, in the middle, and at the end of the trial to evaluate possible alterations of the pattern of endogenous steroids due to the anabolic treatment. The efficacy of the selected implant was verified calculating the feed conversion index of control (9.54) and treated animals (8.51), attesting for increased performances after treatment.

### 3.2. Analytical Methods Development

Preliminary experiments were performed to select the conditions for the triple quadrupole analyzer (API 4000, AB SCIEX, Framingham, MA, USA) and the mobile phase composition, by individually infusing the steroid standards. Acetonitrile with water and methanol with water, in presence of different modifiers such as 0.1% formic acid, or 0.02% ammonium hydroxide, or 5 mM ammonium acetate, both in ESI+ and in ESI− were compared. Acetonitrile with water in presence of 0.02% ammonium hydroxide gave the best result in terms of signal intensity, but, to achieve the desired performances, different ionization techniques were combined. ESI− was chosen for the analysis of estrogens; positive ionization mode was selected to detect androgens, most of which were analyzed in ESI+, whereas APCI+ source was chosen in the case of DHEA, DHT, and ETIO. The latter molecules are characterized by the presence of a keto-group in position 17 and the lack of proximal electron-donor moieties. Thus, to achieve higher signal intensity and lower interference, the APCI source was used as previously reported in the literature [25, 45]. Using such ionization mode, the presence of ammonium hydroxide negatively affected the signal intensity; thus three separate chromatographic and ionization conditions combined with a common purification procedure were used.

During the method development, the best purification conditions were determined comparing C_18_ and HLB SPE cartridges combined with NH_2_ cartridges, to reduce at most the matrix effect and ensure a satisfactory recovery for the analyzed molecules. C_18_ (500 mg, 6 mL) and HLB (200 mg, 6 mL) SPE were tested in parallel: SPE activation conditions and loading volumes of sample extracts were the same for both of them. Different percentages of methanol/water (25/75, 50/50, and 75/25, v/v) solutions were tested for the washing step. An acceptable compromise between drug recovery and removal of interfering compounds was found when coupling HLB to NH_2_ cartridges using methanol/water (50/50, v/v) mixture: when higher percentage of methanol was chosen, recoveries of more polar compounds worsened. Similarly, also different solutions of elution were tested in parallel on both SPE: best recoveries were achieved with the proposed combination of methanol and ethyl acetate.

For deconjugation of steroids glucuronides and sulfates we used two different techniques.* Helix pomatia* juice containing arylsulphatase and* Helix pomatia* juice containing *β*-glucuronidase that are commonly used in many screening and confirmatory methods [8, 46] were employed for the detection of the administered sexual steroids and their epimers. The choice of using two* Helix pomatia* juices with specific activity towards sulfated and glucuronated conjugates is necessary to quantify the total concentration of steroids, without need to analyze each sample for steroids content both in their free form and as conjugates.

For the analysis of endogenous steroids, a combination of *β*-glucuronidase produced in* E. coli* followed by methanolysis in acidic conditions [47] was used. Indeed, previous studies demonstrated that the use of* Helix pomatia* juice can produce misleading results due to the presence of other enzymes that are responsible for steroids conversion [48, 49] that could affect the correct determination of 17*α*-T, AED, DHEA, DHT, and ETIO. This different approach was used for the secondary goal of performing multivariate analyses based on the urinary profile of endogenous steroids to develop a probabilistic model able to discriminate treated animals from nontreated ones. Accordingly, urine samples collected at the beginning, in the middle, and at the end of the* in vivo* trial (i.e., *T*_0_, *T*_35_, and *T*_68_) were also processed using this alternative deconjugation method, to specifically quantify E1, 17*β*-T, 17*α*-T, AED, DHEA, DHT, and ETIO.

**Figure 1:**
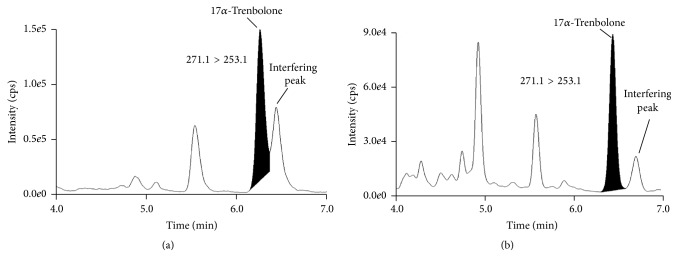
Representative SRM chromatograms for 17*α*-TBOH. (a) shows the most intense transition (271.1 > 253.1) monitored using organic mobile phase not containing methanol, while (b) shows the same transition acquired in presence of methanol in the organic phase (acetonitrile/methanol 70/30, v/v). In both SRM chromatograms the peak of 17*α*-TBOH is integrated and the interfering peak physiologically present in bovine urine is indicated.

The selection of the chromatographic conditions was performed comparing the SRM spectra from blank urine samples and spiked ones. Using acetonitrile and water with 0.02% ammonium hydroxide as mobile phase, a peak showing the same transitions of 17*α*-TBOH (271.1 > 253.1, 271.1 > 199.1) was observed around the same retention time in blank urine. Different gradient elution and isocratic conditions were tested and compared to achieve a separation of the interfering peak from 17*α*-TBOH. Finally, these two different compounds were successfully separated using a slow gradient (Table 2) and a mobile phase composed of acetonitrile/methanol (70/30, v/v) without the ammonium hydroxide that was maintained only in the aqueous solution. By means of this organic mobile phase the use of immunoaffinity purification was not required (Figure 1).

### 3.3. Validation Study

The reported LC-MS/MS methods were developed and validated according to Commission Decision 2002/657/EC, properly adapted for endogenous steroids.

**Figure 2:**
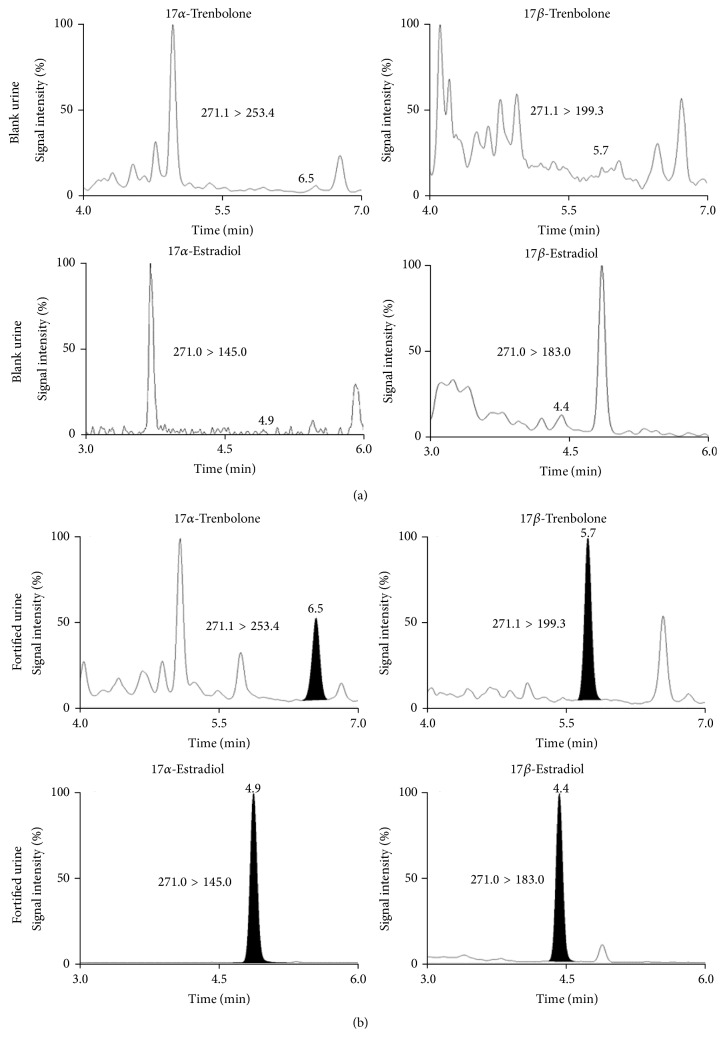
SRM chromatograms of the target drug residues and their main metabolites. In (a) a blank urine sample is reported, and in (b) the same sample spiked at the lowest level of the calibration curve constructed in urine for the exogenous molecules and in charcoal-depleted urine for 17*α*-E2.

**Figure 3:**
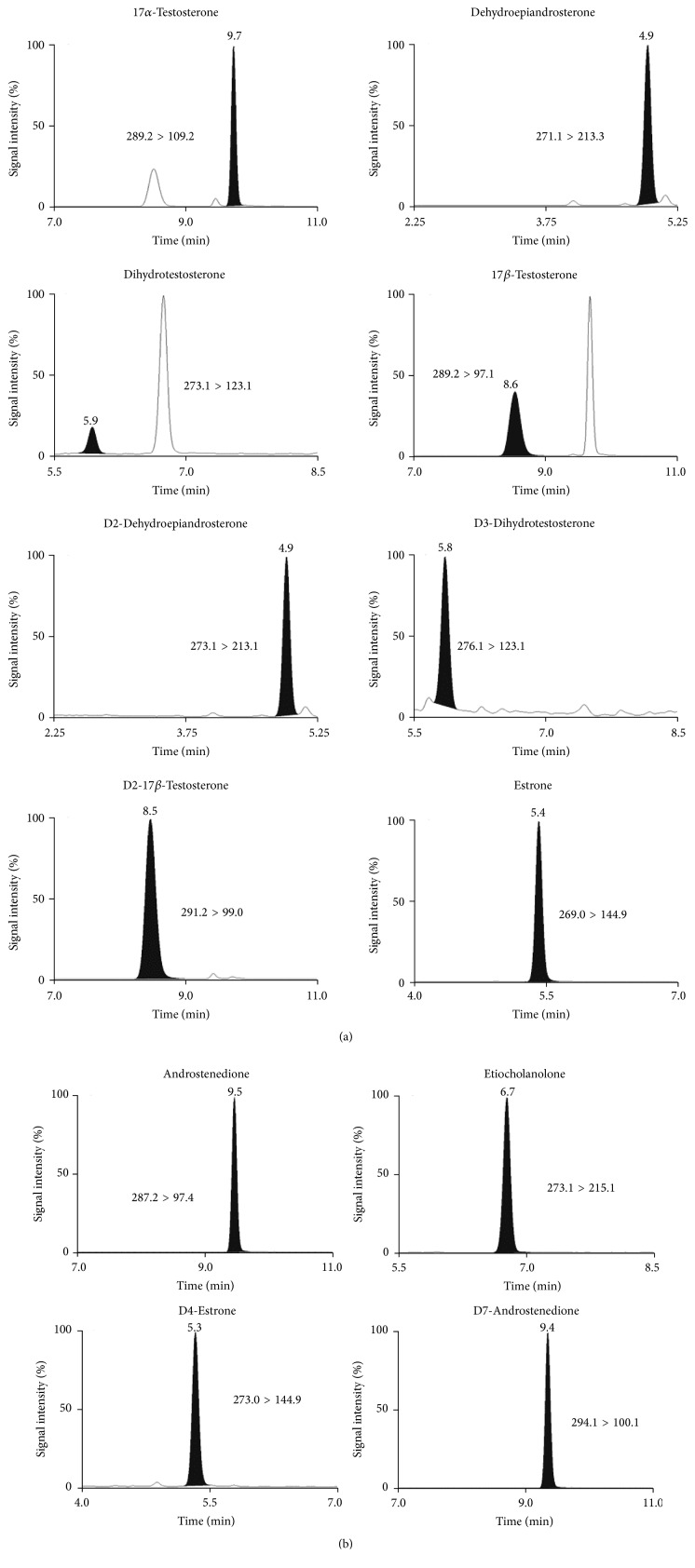
SRM chromatograms of the target steroids spiked at the lowest level of the calibration curve constructed in charcoal-depleted urine. The retention time and monitored transitions are reported for each analyte.

The specificity of the methods was verified analyzing 20 blank bovine urine samples for the exogenous substances and 20 blank charcoal-depleted urine samples for the endogenous compounds. No interfering peaks were found at the expected retention time of the target analytes. In particular, an endogenous peak showing the same transitions as 17*α*-TBOH but with an altered ion ratio was found to be present. This peak did not interfere with the quantification of 17*α*-TBOH residues because the chromatographic separation was acceptable (Figure 1). Linearity of the response was verified with 5 calibration levels, including a blank urine sample, using a lack of fit test. The curve was injected at the beginning of each batch of analysis. Independent calibration curves were built in each of the three separate working sessions, during method validation performed on fortified samples (Table 3). The correlation coefficients (Pearson *R*) of the standard curves were found to be better than 0.996. The standard calibration curves were prepared in urine for the quantification of exogenous substances and in charcoal-depleted urine for the endogenous steroids. Representative SRM chromatograms for target drug residues and their main metabolites at the lowest calibration curve level are shown in Figure 2. The SRM chromatograms of the other analyzed compounds are reported in Figure 3.

During method validation for the exogenous molecules (17*α*-TBOH, 17*β*-TBOH, and 17*β*-E2), seven blank urine samples fortified with the steroids at four concentration levels (Table 3) were prepared and analyzed in three different working sessions by two different operators. The same approach was applied for the endogenous steroids (17*α*-E2, E1, 17*β*-T, 17*α*-T, AED, DHEA, DHT, and ETIO), using charcoal-depleted urine. The validation range for endogenous compounds was preliminarily defined analyzing 10 urine samples collected from bulls under field conditions and taking into account instrument sensitivity. For all the molecules under investigation the recovery was corrected by a deuterated internal standard. ETIO was quantified using D2-DHEA to correct for recovery. The deuterated-*β*-isomer form of a given steroid was used to correct for both the *α*- and the *β*-epimer. Because *α*-isomers and deuterated-*β*-isomers are not eluted at the same retention time, there may be no compensation of the matrix effects. In these cases, our results showed an acceptable recovery ranging from 81.6% for ETIO to 110.0% for 17*α*-TBOH, displaying an intermediate precision (CV%) lower than 15% for all the tested molecules.

Decision limit (CC*α*) and detection capability (CC*β*) were calculated according to Commission Decision 2002/657/EC (for forbidden substances) by the ISO 11843 calibration curve procedure following the approach proposed by Verdon et al. and by Lega et al. [50, 51]. The CC*α* values together with the CC*β* for the investigated molecules are reported in Table 3.

Six blank urine samples were fortified at the calculated CC*α* with 17*α*-TBOH, 17*β*-TBOH, and 17*β*-E2 and analyzed to verify the reliability of the estimated decision limits. In all samples each compound was correctly detected and quantified, attesting for acceptable method performance even below extrapolated CC*β*.

Youden test was adopted to verify method ruggedness to minor changes [52]; seven variables (i.e., pH, deconjugation time and temperature, washing solution composition during SPE purification, elution solvent composition, mobile phase composition, and evaporation temperature) were modified with respect to the prescribed procedure to evaluate methods ruggedness to minor changes. The quantitative results were not significantly different compared to those obtained employing the standard conditions, proving that limited variations (±5%) of the chosen variables do not affect the method performance.

### 3.4. Urinary Steroids Profile

**Figure 4:**
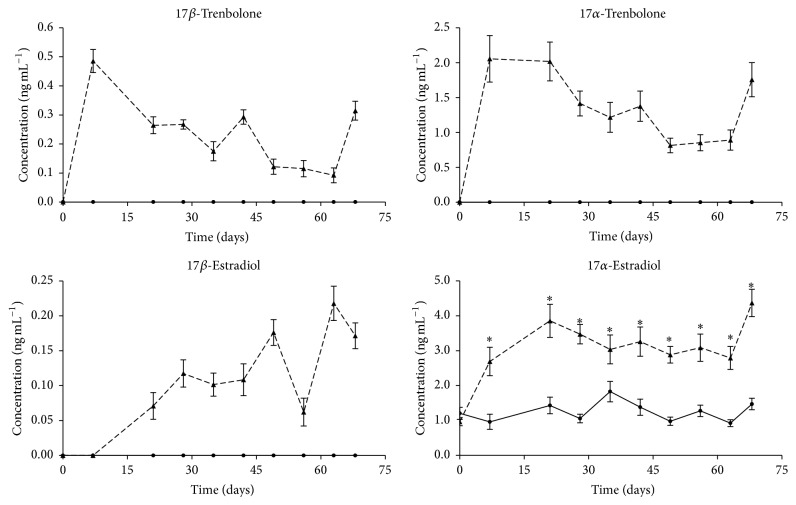
Time dependent excretion profile for 17*α*-TBOH, 17*β*-TBOH, 17*β*-E2, and 17*α*-E2 in urine samples from bulls treated with the ear implant (dashed line) and untreated bulls (solid line). Values are reported as mean ± SEM (*n* = 16 for each animal group). ^*∗*^ Student's *t*-test *p* value < 0.05.

**Figure 5:**
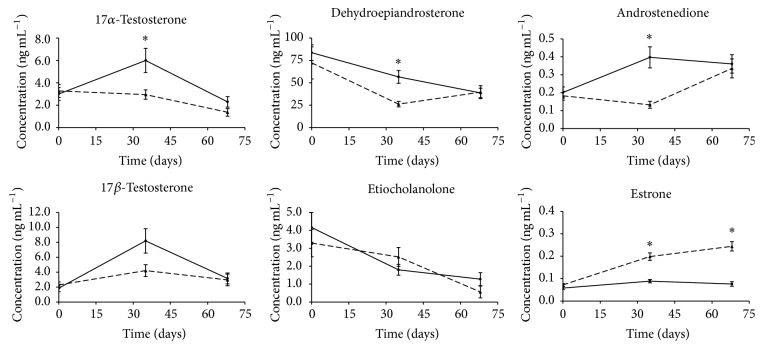
Time dependent excretion profile of endogenous steroids physiologically present in urine samples from bulls treated with the ear implant (dashed line) and untreated bulls (solid line) gathered during the trial. Concentration values were measured at times *T*_0_, *T*_35_, and *T*_68_ (before, in the middle, and at the end of the treatment) using *β*-glucuronidase from* E. coli *followed by solvolysis in acidic conditions for deconjugation of analytes (see materials and methods for details). Reported values are mean ± SEM (*n* = 16 for each animal group). ^*∗*^ Student's *t*-test *p* value < 0.05.

To draw the excretion profile during the treatment period (Figures 4 and 5), all 32 urine samples at each sampling day were analyzed in duplicate. Mean concentration values and standard errors are summarized in Table 4. For such purpose, we included in the calculations all the analytical values above CC*α*: for those below the decision limit, a zero value was assigned. When calculated mean concentrations were below CC*β*, they were reported in italic for reader information. Data were not normalized to creatinine or specific density, such approaches being not yet fully accepted as reference standards and considering the good precision of collected data.

The urinary excretion profile of 17*α*-TBOH, 17*β*-TBOH, 17*α*-E2, and 17*β*-E2 was monitored during the entire trial (Figure 4). No drug residues of the exogenous compounds were detectable in urine from control animals. Previous research studies conducted to reproduce putative illicit veterinary practices were performed administering anabolic steroids by intramuscular injection [28, 42–44]. In such conditions high concentrations of target compounds or their metabolites were recorded immediately after injection and were rapidly excreted within few days requiring repeated injections to achieve the anabolic effect. Conversely, the ear implant used in the present study slowly released its content, showing low residual urinary concentration of 17*β*-TBOH and 17*β*-E2 throughout the whole treatment period. The excretion profile is significantly different from the typical excretion kinetic observed after intramuscular injection.

17*α*-TBOH and 17*β*-TBOH were found to be present in urine starting from the initial stage of the trial. They already reached their maximum concentration on the first sampling point, displaying urinary levels that decreased slowly during the following 63 days of treatment. At *T*_63_, the concentration was almost halved compared to *T*_7_. This time point is very close to the moment in which the uncoated part of the ear implant is supposed to terminate the drug release, while the coated part should begin its degradation allowing the delayed release of its content (according to the manufacturer's instructions). This fact is confirmed by data collected at *T*_68_ which showed a slight increase in 17*α*-TBOH and 17*β*-TBOH level and by the similar behavior observed for 17*β*-E2 between *T*_56_ and *T*_63_ (Figure 4). On the other hand 17*β*-E2 behaves in a different way with respect to TBOH, especially at the beginning of the trial (*T*_7_), where all the treated samples display concentrations well below the CC*α*. Indeed its presence can only be indirectly hypothesized thanks to the comparison of the urinary concentration of its metabolite 17*α*-E2 that is significantly higher in T samples with respect to C samples at *T*_7_ (Figure 4). Conversely, from *T*_21_, 17*β*-E2 concentration showed a slow increase until *T*_49_. It is also important to note that, at each sampling point throughout the trial, one-third of the treated samples showed 17*β*-E2 concentration below the calculated CC*α*, confirming that the tested device may reproduce a putative anabolic treatment based on slow release of steroids.

Interestingly, these data fit well with the results achieved by Blackwell et al. [20] in a similar study where a group of 8 steers received Revalor-XS implants and were followed over a period of 112 days. The urinary concentration of 17*α*-TBOH was very similar in absolute concentration and trend over the same period of time. The global picture of urinary excretion of 17*α*-E2 is also similar, considering the different precision of the methods, and both experiments showed significantly different 17*α*-E2 concentrations between implanted and nonimplanted animals. Similar data on urinary concentration of 17*β*-TBOH and 17*β*-E2 are not reported in Blackwell's work, but they can be inferred from data in serum exhibiting similar trends.

Urine samples collected at the beginning, in the middle, and at the end of the* in vivo* trial (i.e., *T*_0_, *T*_35_, and *T*_68_) were used for the secondary goal of performing multivariate analyses based on the urinary profile of endogenous steroids to develop a probabilistic model able to discriminate treated animals from nontreated ones. The urinary concentration of the seven monitored endogenous steroids is reported in Figure 5; DHT is not reported because its urinary concentration was too low to be consistently quantified, whereas urinary ETIO values are reported, but the measured concentrations are possibly not accurate, the observed values being lower than the validation range. Where average concentrations of each analyte were statistically different between T and C groups, they were indicated by asterisks (Student's *t*-test *p* value < 0.05).

The analysis of E1 pointed out a significant difference between T and C animals that was maintained for the entire trial, as also for 17*α*-E2, indicating that these metabolites of 17*β*-E2 are very important for the indirect detection of illicit treatments with E2, as reported in previous studies [17, 42, 44]. Statistically significant different concentrations of estrone between implanted and nonimplanted animals were observed by Blackwell et al. [20], though in this case the paper does not report E1 excretion during the entire trial. Differences in absolute concentrations observed between these two studies might depend on the animals (steers rather than bulls), breed, and the use of the appropriate internal standard (deuterated E1) that was applied in the present paper for E1 quantification. AED, 17*α*-T, and DHEA were found to be significantly reduced in T animals compared to C animals only at *T*_35_. At *T*_68_ the observed difference was no more appreciable (Figure 5).

A multivariate logistic regression analysis was performed using software R taking into account concentration values of endogenous steroids (i.e., 17*α*-E2, E1, 17*β*-T, 17*α*-T, AED, DHEA, and ETIO). Measured concentrations at *T*_0_, *T*_35_, and *T*_68_ were used to derive the regression equation by forward stepwise selection of variables used as threshold for inclusion of *p* < 0.05. The significant variables to be included in the model resulted in being DHEA and E1. Coefficients, standard error, and *p* values are reported in Table 5.

**Figure 6:**
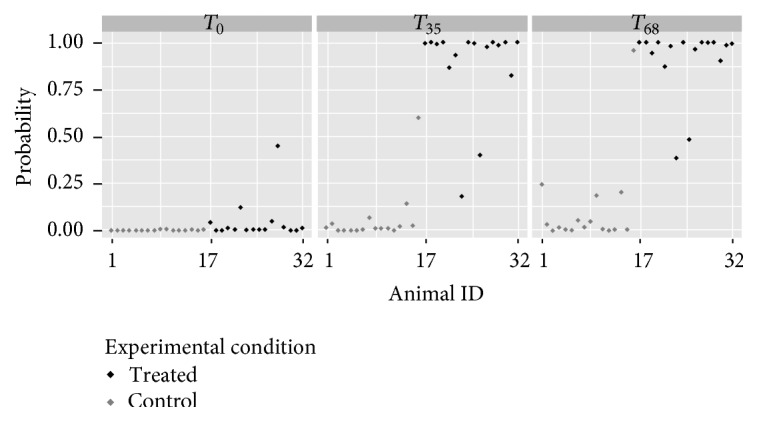
Estimated probability of being classified as treated animal, based on logistic regression model with DHEA and E1 as predictors. Urine samples were collected to measure endogenous steroids profiles at times *T*_0_, *T*_35_, and *T*_68_ (before, in the middle, and at the end of the treatment) from 32 animals divided into two groups: in grey control animals identified with animal ID from 1 to 16 and in black treated animals identified with animal ID from 17 to 32.

Concentration values of these two endogenous steroids were used to determine the probability of classifying an animal as treated, defined as

(1) $$\begin{array}{c} P\left(\;Y=1\;\right)=\frac{1}{1+e^{-z}}, \end{array}$$

where *Y* = 1 for treated animals and *z* = 5.805 × [E1] − 4.449 × [DHEA] − 2.620. The interpretation of coefficients in terms of odds ratio (OR) is that an increase of one unit of E1 results in a change of the log odds of being classified as treated of 5.805. The predicted probability for all the animals is illustrated in Figure 6.

The ability of the model to correctly classify animals can be assessed by computing the ROC curve, evaluating the performance of the model at all possible cut points. The area under the calculated ROC curve was 0.9902 (95% CI: 0.9775–1) indicating a good predictive ability.

## 4. Conclusions

The first purpose of our study was to monitor the residual urinary concentration of 17*β*-TBOH and 17*β*-E2 and their main metabolites 17*α*-TBOH and 17*α*-E2 after application of an implant delivering low concentrations of TBA and 17*β*-E2. The developed multiresidue method is suitable for most considered compounds but, in perspective, it should be improved to achieve lower CC*α* and CC*β* particularly for 17*β*-E2 and 17*β*-TBOH to guarantee accurate measurements at sub-ng mL^−1^ levels. The calculated concentrations for 17*α*-TBOH and 17*α*-E2 were in the range of 1–5 ng mL^−1^, while for 17*β*-TBOH and 17*β*-E2 they were found to be between 0 and 0.5 ng mL^−1^. Data collected confirm the results from previous studies where the same type of anabolic treatment was adopted. The low concentrations observed for these target analytes during the whole considered period confirm that the experimental treatment adopted in this study produces a totally different urine excretion profile with respect to the effect produced by intramuscular injection and should recommend adopting very sensitive analytical protocols to detect and quantify these residues in urine for official controls.

The secondary target of this work was the evaluation of the urinary profile of a set of endogenous steroids to verify their potential as indirect biomarkers, taking advantage of the applicability of the same analytical protocol, slightly modified. The concentrations measured for the analyzed endogenous steroids indicated that the adopted implant partially alters the profile of some endogenous steroids without affecting considerably the status of the animal. In this regard, an alternative approach for the indirect detection of anabolic treatment was also explored combining the concentration of DHEA and E1 measured in each animal group, giving a promising result of correct predicted assignment of 93.7% and a group misallocation of 6.3%, with a cut point of 0.5.

The combination of these results with those derived from other studies applied to the same animals [29, 33, 53] indicates that an integrated combination of residue analyses, targeted metabolic profiles, and untargeted metabolomics and proteomics approaches represents the state of the art for a more powerful strategy to control and possibly inhibit illicit practices of growth promotion.

## Figures and Tables

**Table 1:** Instrumental settings optimized for the analyzed compounds.

Steroid	Ionization mode	Retention time (min)	SRM transition (Q1/Q3)	Declustering potential	Collision energy
DHEA	APCI+	4.9	271.1/213.3	60	25
			271.1/81.2	60	43
D2-DHEA	APCI+	4.9	273.1/213.1	60	24
DHT	APCI+	5.9	273.1/123.1	70	28
			291.1/255.1	70	19
D3-DHT	APCI+	5.8	276.1/123.1	70	33
ETIO	APCI+	6.7	273.1/215.1	70	24
			273.1/159.0	70	31

17*α*-TBOH	ESI+	6.5	271.1/253.4	70	31
			271.1/199.3	70	36
17*β*-TBOH	ESI+	5.7	271.1/199.3	70	36
			271.1/227.3	70	31
D3-17*β*-TBOH	ESI+	5.6	274.1/199.1	70	35
17*α*-T	ESI+	9.7	289.2/109.2	60	35
			289.2/97.1	60	35
17*β*-T	ESI+	8.6	289.2/97.1	60	35
			289.2/109.2	60	35
D2-17*β*-T	ESI+	8.5	291.2/99.0	60	35
AED	ESI+	9.5	287.2/97.4	60	34
			287.2/109.1	60	36
D7-AED	ESI+	9.4	294.1/100.1	60	35

17*α*-E2	ESI−	4.9	271.0/145.0	−100	−54
			271.0/239.0	−100	−53
17*β*-E2	ESI−	4.4	271.0/183.0	−100	−54
			271.0/145.0	−100	−54
D4-17*β*-E2	ESI−	4.4	275.0/145.0	−100	−75
E1	ESI−	5.4	269.0/144.9	−100	−55
			269.0/142.9	−100	−75
D4-E1	ESI−	5.3	273.0/144.9	−100	−75

**(a):** 

APCI+	A1 (%)	B1 (%)
Time (min)	H_2_O	ACN
0.00	70.0	30.0
1.00	70.0	30.0
1.01	62.5	37.5
6.00	50.0	50.0
6.50	10.0	90.0
7.50	10.0	90.0
8.00	70.0	30.0
10.50	70.0	30.0

**(b):** 

ESI+	A2 (%)	B2 (%)
Time (min)	H_2_O + 0.02% NH_4_OH	ACN/MeOH (70/30)
0.00	70.0	30.0
1.00	70.0	30.0
1.01	65.0	35.0
7.00	62.5	37.5
7.01	25.0	75.0
8.50	25.0	75.0
8.75	10.0	90.0
9.75	10.0	90.0
10.25	70.0	30.0
12.75	70.0	30.0

**(c):** 

ESI−	A3 (%)	B3 (%)
Time (min)	H_2_O + 0.02% NH_4_OH	ACN + 0.02% NH_4_OH
0.00	70.0	30.0
1.00	70.0	30.0
1.01	62.5	37.5
6.00	50.0	50.0
6.50	10.0	90.0
7.50	10.0	90.0
8.00	70.0	30.0
10.50	70.0	30.0

**(a):** 

	17*α*-TBOH				17*β*-TBOH				17*α*-T				17*β*-T			
Validation level (ng mL^−1^)	0.5	0.75	1.0	2.5	0.5	0.75	1.0	2.5	2.5	5.0	20.0	35.0	1.0	2.0	8.0	14.0
Calibration level (ng mL^−1^)	0.3	1.0	2.0	3.0	0.3	1.0	2.0	3.0	3.0	20.0	40.0	60.0	1.2	8.0	16.0	24.0
Mean recovery (%)	110.0	108.1	105.0	104.2	102.8	102.6	101.2	104.3	97.9	98.0	98.2	99.6	103.7	92.8	99.6	100.6
Intermediate precision (CV%)	6.8	6.8	8.5	9.4	4.9	4.5	7.2	7.1	7.6	8.0	9.3	8.4	4.6	7.5	3.6	3.7
CC*α* (ng mL^−1^)	0.23				0.17				1.42				0.55			
CC*β* (ng mL^−1^)	0.39				0.29				2.41				0.93			

**(b):** 

	AED				DHEA				DHT				ETIO			
Validation level (ng mL^−1^)	0.3	0.5	1.0	1.5	2.0	5.0	10.0	15.0	2.0	5.0	10.0	15.0	20.0	50.0	100.0	150.0
Calibration level (ng mL^−1^)	0.2	1.0	2.0	3.0	3.0	10.0	20.0	30.0	3.0	10.0	20.0	30.0	30.0	100.0	200.0	300.0
Mean recovery (%)	101.7	88.4	93.6	94.1	109.8	99.9	97.5	106.6	94.1	95.8	97.4	100.4	101.1	81.6	91.2	104.1
Intermediate precision (CV%)	3.1	7.6	3.4	4.8	12.3	14.1	13.5	11.9	12.4	7.7	6.1	6.6	2.7	6.9	6.5	11.2
CC*α* (ng mL^−1^)	0.08				2.29				1.09				18.62			
CC*β* (ng mL^−1^)	0.14				2.51				1.85				31.68			

**(c):** 

	17*α*-E2				17*β*-E2				E1			
Validation level (ng mL^−1^)	0.5	1.0	1.5	2.5	0.5	0.75	1.0	2.5	0.1	0.4	0.7	1.0
Calibration level (ng mL^−1^)	0.3	1.0	2.0	3.0	0.3	1.0	2.0	3.0	0.06	0.4	0.8	1.2
Mean recovery (%)	91.4	96.9	99.1	101.2	98.2	97.7	100.9	103.3	91.0	97.5	98.6	96.4
Intermediate precision (CV%)	8.0	6.1	4.0	4.8	11.6	7.4	6.5	5.7	9.7	4.4	4.0	4.2
CC*α* (ng mL^−1^)	0.13				0.15				0.05			
CC*β* (ng mL^−1^)	0.22				0.26				0.08			

**(a):** 

	17*α*-TBOH [ng mL^−1^]		17*β*-TBOH [ng mL^−1^]		17*α*-E2 [ng mL^−1^]		17*β*-E2 [ng mL^−1^]	
	CNTR	Treated	CNTR	Treated	CNTR	Treated	CNTR	Treated
*T*_0_	n.d.	n.d.	n.d.	n.d.	1.20 ± 0.18	0.96 ± 0.11	n.d.	n.d.
*T*_7_	n.d.	2.06 ± 0.33	n.d.	0.49 ± 0.04	0.96 ± 0.22	2.69 ± 0.41	n.d.	n.d.
*T*_21_	n.d.	2.02 ± 0.28	n.d.	0.26 ± 0.03	1.43 ± 0.24	3.85 ± 0.47	n.d.	0.07 ± 0.02
*T*_28_	n.d.	1.42 ± 0.18	n.d.	0.27 ± 0.02	1.05 ± 0.12	3.47 ± 0.28	n.d.	0.12 ± 0.02
*T*_35_	n.d.	1.22 ± 0.21	n.d.	0.20 ± 0.03	1.82 ± 0.29	3.03 ± 0.41	n.d.	0.10 ± 0.02
*T*_42_	n.d.	1.38 ± 0.22	n.d.	0.29 ± 0.02	1.38 ± 0.23	3.25 ± 0.42	n.d.	0.11 ± 0.02
*T*_49_	n.d.	0.81 ± 0.10	n.d.	0.15 ± 0.02	0.97 ± 0.12	2.88 ± 0.24	n.d.	0.18 ± 0.02
*T*_56_	n.d.	0.85 ± 0.12	n.d.	0.15 ± 0.02	1.27 ± 0.16	3.08 ± 0.39	n.d.	0.06 ± 0.02
*T*_63_	n.d.	0.89 ± 0.14	n.d.	0.14 ± 0.02	0.92 ± 0.10	2.79 ± 0.33	n.d.	0.22 ± 0.02
*T*_68_	n.d.	1.76 ± 0.25	n.d.	0.31 ± 0.03	1.47 ± 0.16	4.36 ± 0.39	n.d.	0.17 ± 0.02

**(b):** 

	AED [ng mL^−1^]		DHEA [ng mL^−1^]		E1 [ng mL^−1^]		ETIO [ng mL^−1^]		17*α*-T [ng mL^−1^]		17*β*-T [ng mL^−1^]	
	CNTR	Treated	CNTR	Treated	CNTR	Treated	CNTR	Treated	CNTR	Treated	CNTR	Treated
*T*_0_	0.20 ± 0.03	0.19 ± 0.02	83.67 ± 8.74	72.22 ± 17.95	0.06 ± 0.01	0.07 ± 0.01	4.16 ± 0.83	3.29 ± 0.76	3.00 ± 0.58	3.27 ± 0.58	1.86 ± 0.50	2.30 ± 0.40
*T*_35_	0.40 ± 0.06	0.13 ± 0.02	56.57 ± 7.13	26.23 ± 3.12	0.09 ± 0.01	0.20 ± 0.02	1.80 ± 0.30	2.52 ± 0.53	6.00 ± 1.08	2.95 ± 0.42	8.19 ± 1.64	4.20 ± 0.80
*T*_68_	0.36 ± 0.05	0.34 ± 0.05	38.71 ± 5.35	39.54 ± 7.23	0.08 ± 0.01	0.24 ± 0.02	1.28 ± 0.36	0.56 ± 0.33	2.30 ± 0.47	1.37 ± 0.35	3.16 ± 0.73	2.96 ± 0.78

**Table 5:** Coefficients, standard deviations, and *p* values calculated for the variables in the logistic regression model.

	Estimate	SE	*p* value
Intercept	−2.620	1.058	0.01328
DHEA	−4.449	1.729	0.01005
E1	5.805	1.587	0.00025
